# Supplementary material for: Content-rich biological network constructed by mining PubMed abstracts
Source: BMC Bioinformatics. 2004 Oct 8;5:147. doi: 10.1186/1471-2105-5-147 (PMC528731; doi:10.1186/1471-2105-5-147)
Supplement: Additional File 5 — The original Chilibot query results of the term "long-term potentiation (LTP)" and 22 other terms, limiting the latest references analyzed to the years 1990, 1995, 2000, and 2004. [file 1471-2105-5-147-S5.bz2 › chilibotAdditionalFile5/ltp2004/html/SYNAPSIN I_NMDA.html]

 


 **SYNAPSIN I** and **NMDA** 
  
Found 16 abstracts in PubMed,  **16 abstracts were retrieved and analyzed**.  


---

 Search Google  |
 PDF files only 
|  EDU domain only 

---

**Interactive relationship** (e.g. stimulation, inhibition, etc)

**Parallel relationship** (e.g. studied together, co-existance, homology, etc.)

- **NMDA**  elicited Ca signals were detected only in cells displaying neuronal morphology, but preceding the appearance of  **synapsin I**  immunoreactivity.  Ref: 11413229 J Neurochem, 2001
- Despite the lack of  **NMDA**  receptor function, NR1 neurons formed  **synapsin I**  positive presynaptic boutons associated with MAP2ab positive dendrites in culture.  Ref: 9753105 Eur J Neurosci, 1998
- However, exposure of low glutamine cultures to 100 mM ethanol for 4 days significantly increased the levels of  **NMDA**  receptor subunits and AMPA receptor subunits, but had no effect upon kainate receptor subunits or the synapse associated proteins  **synapsin I**  and PSD 95.  Ref: 10069569 Alcohol Clin Exp Res, 1999
- K 252a had no detectable effect on isolated N methyl D aspartate  **NMDA**  receptor mediated EPSPs but significantly inhibited the in situ phosphorylation of specific hippocampal proteins  **synapsin I** , MARCKS, and B 50.  Ref: 10467568 Learn MemLearn Mem, 1999
- The formation of functional  **NMDA**  receptors also coincided with the appearance of  **synapsin I**  and synaptophysin.  Ref: 11920728 J Neurobiol, 2002
- These results suggest that glutamate can activate CaM kinase II through  **NMDA**  receptors in the induction of LTP and in turn stimulates the phosphorylation of target proteins such as MAP2 and  **synapsin I** .  Ref: 8282267 Nippon Yakurigaku Zasshi, 1993
- The effect of N methyl D aspartate  **NMDA**  and 2 aminomethyl phenylacetic acid kainate AMPA kainate glutamate receptors on dentate cell proliferation and hippocampal  **synapsin I**  induction was examined after global ischemia.  Ref: 11129783 J Cereb Blood Flow Metab, 2000
- These results suggest that glutamate can activate CaM kinase II through the ionotropic  **NMDA**  receptor, which in turn increases the phosphorylation of microtuble associated protein 2 and  **synapsin I** .  Ref: 1358879 J Biol Chem, 1992
- **NMDA**  and AMPA kainate glutamate receptors modulate dentate neurogenesis and CA3  **synapsin I**  in normal and ischemic hippocampus.  Ref: 11129783 J Cereb Blood Flow Metab, 2000
